# Adrenocortical Carcinoma: Updates of Clinical and Pathological Features after Renewed World Health Organisation Classification and Pathology Staging

**DOI:** 10.3390/biomedicines9020175

**Published:** 2021-02-10

**Authors:** Alfred King-yin Lam

**Affiliations:** 1 School of Medicine, Griffith University, Gold Coast, QLD 4222, Australia; a.lam@griffith.edu.au; 2 Pathology Queensland, Gold Coast University Hospital, Southport, Gold Coast, QLD 4215, Australia; 3 Faculty of Medicine, The University of Queensland, Herston, Brisbane, QLD 4006, Australia

**Keywords:** adrenocortical carcinoma, adrenal, staging, pathology, oncocytic, myxoid, sarcomatoid

## Abstract

Adrenocortical carcinoma (ACC) is a heterogenous group of diseases with different clinical behaviour between adult and paediatric patients. In addition, three histological variants, oncocytic, myxoid and sarcomatoid are noted on the recent World Health Organisation (WHO) classification of ACC. A review of recent literature showed that the different types of ACC have distinctive demographic data, clinical presentation, pathology, biological behaviour, genomic and patients’ prognosis. In addition, recent updates of pathology staging for ACC allow refinement of prognostic grouping for planning treatment of the patients with ACC. These advances in genomic, pathology and staging have driven the development of standardisation of pathology reporting. International standardisation of pathological reporting of adrenocortical carcinoma and adaption to local pathology communities provide universal platforms for clinicians and researchers involved in the management of patients with ACC. To conclude, all these advances in the field of pathology will improve development of management strategies including improvement of clinical care, development of prognostic markers and testing of novel therapeutic approaches for patients with adrenocortical carcinoma.

## 1. Introduction

Adrenal cortical carcinoma (ACC) is a rare cancer but is the most common primary cancer in the adrenal gland [1]. It is the second most common malignant tumour of the endocrine organ after anaplastic thyroid carcinoma [2]. In large pathology series, ACC accounts for 6.8% of all primary adrenal tumours [3]. In the most recent population database (based on approximately 2000 cases) from the Surveillance, Epidemiology, and End Results (SEER) published in 2018 from the United States of America (USA) of ACC-diagnosed patients between 1973 to 2012, the annual incidence of the cancer was 1.02 per one million [4]. This figure has reflected the increase incidence of the cancer due to increased use of sensitivity imaging studies in current years. In addition, in a recent large series of adrenal incidentalomas (*n* = 3672), ACC accounts for 1.4% of cases [5].

There is an increase of new knowledge about ACC in terms of genomic characterisation based on the findings of The Cancer Genome Atlas (TCGA) [6,7,8,9]. Further genomic studies showed that mutational and expression profiles of advanced and metastatic ACC are very similar to those from primary ACC as well as low mutation rates, few major oncogenic drivers and loss of function mutations in several epigenetic regulators suggest an epigenetic basis of ACC such as DNA methylation [10,11]. In a similar period, the eighth American Joint Committee on Cancer (AJCC) Cancer Staging Manual and UICC (Union for International Cancer Control) Tumour, Lymph node, Metastasis (TNM) staging Manual published in 2016 (adopted for use in 2017) have updated the pathological staging of ACC based on the European Network for the study of Adrenal Tumours (ENSAT) [12]. In addition, the fourth edition of the World Health Organization (WHO) book series on tumour classification published in 2017 has updated the histological classification of ACC [13].

The present review focuses on knowledge about these advances in the pathological field as well as updates of current advances of the ACC. In the 4th (current) WHO classification of tumours of the endocrine system, apart from the conventional ACC, three histological variants of ACC are proposed for the first time in WHO classification [13]. Emphases on the updates are on the unique clinical and pathological features of conventional as well as new histological variants accepted by the WHO classification of tumours of endocrine system.

## 2. Conventional Adrenocortical Carcinoma

### 2.1. Demographic Characteristics

Based on the small number of cases of other histological variants of ACC, conventional ACC comprises more than 90% of cases. Thus, data noted in epidemiological studies likely represent the features of conventional ACC. Like other endocrine tumours, ACC is more common in females. From the pooled series of different countries, ACC is more common in females with male to female ratio of approximately 1 to 1.4 [3,14,15]. The average age at presentation of patients with ACC in larger series range from 47- to 55-year-olds [15]. Although some series demonstrate a bimodal age distribution with peaks in the paediatric group and middle age group, SEER data [4] show that there is a steady increase of ACC with increased age peaking at the sixth and seventh decades.

### 2.2. Clinical Features

Approximately half of ACC is functioning with signs and symptoms of hormone(s) secretion [16]. Patients with functioning ACC were younger, more likely to be females and present with metastatic disease. Of functioning tumours, almost half present with signs or symptoms of cortisol excess (Cushing syndrome). The second most common functional presentation was sex hormone secretion accounting for 20% of cases. These sex hormone-producing ACCs are mainly androgens. Rarely, oestrogen secreting/feminising ACC were reported [17,18,19,20,21]. Aldosterone secreting ACC is uncommon and accounted for approximately 8% of cases. In addition, mixed hormone production was seen in 15% to 25% of functioning ACCs [15,22].

Non-functioning ACCs commonly present with abdominal mass, abdominal pain as well as general symptoms of malignancy. Paraneoplastic manifestations of having ACC such as hypoglycaemia (due to insulin growth factor 2 [IGF2] production) [23] and clinical manifestations related to adrenocorticotropic hormone ACTH production have been reported [24]. Rare manifestations of patients with ACC include cancer-related thrombotic microangiopathy [25] and tumour rupture with retroperitoneal haemorrhage [26,27,28].

ACC could be a cancer in the setting of several hereditary syndromes. These syndromes could occur in high as 5–10% of patients with ACC [29]. In adult patients with ACC, these syndromes include multiple endocrine neoplasia type 1 (*MEN1*, approximately 20 cases reported) [30], Lynch syndrome (mismatch repair genes) (approximately 10 cases) [31], Li-Fraumeni syndrome (*TP53*, more than 10 cases) [32] and neurofibromatosis type 1 (*NF1*, approximately 10 cases) [33]. Rarely, ACC can occur in patients with Carney complex (*protein kinase A regulatory subunit 1A* [*PRKARIA*]) [34,35], Gardner’s syndrome [36] and familial adenomatous polyposis (*adenomatous polyposis coli* [*APC*]) [37,38]. Although extremely rare, ACCs have been reported in patients with congenital adrenal hyperplasia and myelolipoma [39,40,41]. It is likely these are collision tumours rather than having been aetiologically related.

### 2.3. Pathology

ACC is often noted on left adrenal (left to right ratio = 1.2 to 1). Bilateral ACC is uncommon and accounted for approximately 1% of the cases [4]. Ectopic ACCs, presumably arise from ectopic adrenal rests, have been reported in the retroperitoneum [42], ovary [43,44], spinal region [45], liver [46] and abdominal wall [47]. A case of retroperitoneal ACC was noted in 68-year-old man with Lynch syndrome [48].

**Figure 1:**
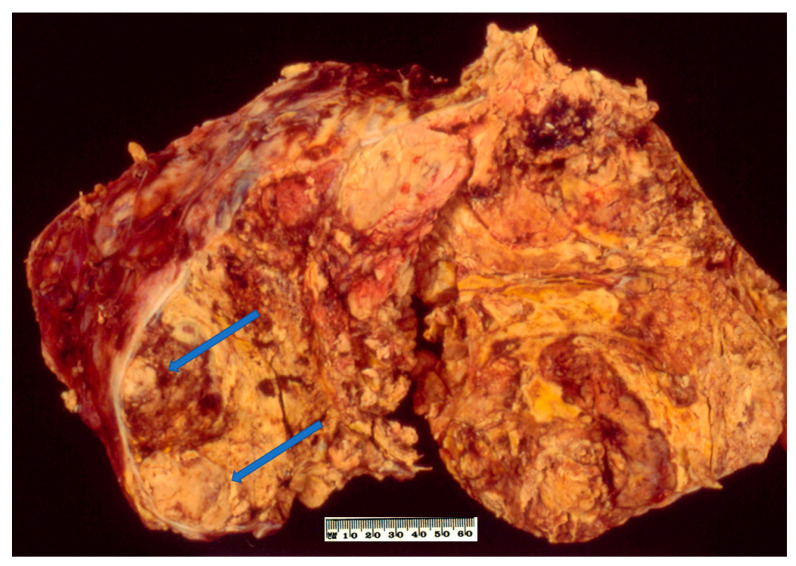
Conventional adrenocortical carcinoma (ACC) showing variegated cut surface of yellow tumour with foci of haemorrhages and necrosis (arrows).

On macroscopic examination, ACC is often yellow to tan colour (Figure 1). Areas of necrosis and haemorrhage are common which give a heterogenous appearance on cut sections. The tumour is often large with median maximum dimension of 100 mm to 120 mm [4,49]. From the findings in recent literature, the largest non-functioning ACC was reported in a 39-year-old Greek woman with a maximum dimension of 237 mm [50] whereas the largest functioning (androgen-secreting) ACC was noted in a 48-year-old Canadian woman with a maximum dimension of 230 mm [51]. Also, Weiss and colleagues have mentioned an ACC with 280 mm in dimension in their series published in 1989 [52] but no clinicopathological details about the case. The tumour could be seen to grossly extend to adjacent organs as well as to regional veins and the right atrium which could lead to pulmonary embolism [53].

**Figure 2:**
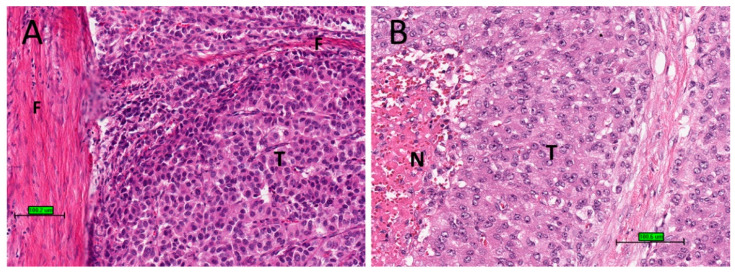
Histological features of ACC. (**A**) Tumour cells (T) with eosinophilic cytoplasm with thick fibrous bands (F). (**B**) Tumour cells (T) with necrosis (N) (haematoxylin and eosin stain) (scale bar–100 µm).

Upon microscopic examination, ACC often has eosinophilic cytoplasm. There are often thick fibrous bands and capsules (Figure 2A). Necrosis is common (Figure 2B). Mitotic figures are often prominent. For prognostic purposes, ACC was subdivided into low grade or high grade depending on mitotic frequency (dividing line is between ≤20 and >20 mitosis per 50 high power fields/10 mm^2^) [52].

It is often not difficult to differentiate ACC from benign cortical adenoma or borderline cortical tumour. Nevertheless, in some instances, the diagnosis of ACC needs to rely on assessment of multiple pathological parameters. The most widely accepted system adopted by WHO classification for assessment is Weiss criteria published in 1984 [52,54]. Using this system, ACC could be diagnosed on at least three of the 9 histological features—high nuclear grade (Fuhrman III or IV), high mitotic rate (>5 mitoses per 50 high power field, atypical mitotic figures, ≤25% clear cells, diffuse architecture, tumour confluent necrosis, venous invasion, sinusoidal invasion, and capsular invasion. Modified Weiss has been proposed which is based on 5 of the 9 histological features of Weiss (mitotic counts, clear cells, atypical mitotic figures, tumour necrosis and capsular invasion) [55]. In this system, mitotic rate and clear cells have twice the points (‘weight”) compared with other features. The outcome of use of modified Weiss is highly correlated with the original Weiss system [55].

Diffuse architecture must be present in >33% and could be identified by disruption of the reticulin [56] and interruption of basal lamina (antibodies to laminin or collagen type IV). A reticulin algorithm has been used for diagnosis of ACC which involves an abnormal/absent reticulum framework and at least one of the 3 of histological features (tumour necrosis, presence of venous invasion and mitotic rate of >5/50 high power field) [56,57].

**Figure 3:**
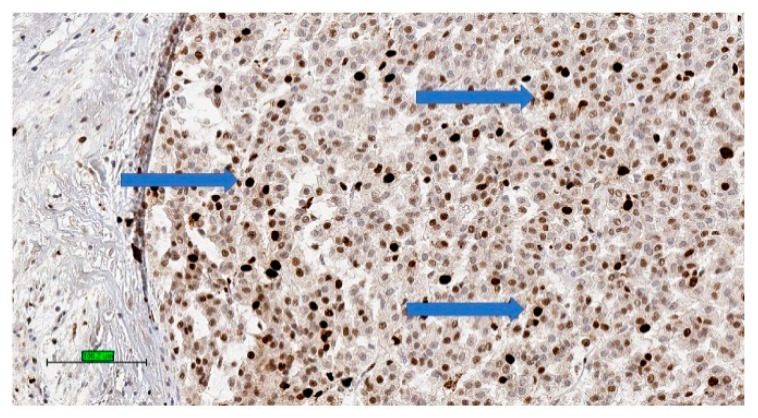
Adrenocortical carcinoma of high Ki-67 index with high percentage of nuclear stain (brown colour) for Ki-67 (3,3′-diaminobenzidine/haematoxylin stains) (blue arrows) (scale bar—100 µm).

These systems for predicting malignant potential of adrenocortical tumours are applicable mainly in conventional ACC. It is important to note that no single microscopic criterion on its own is indicative of malignancy and there is subjective variability in the interpretation [58]. Studies have proposed the use of proliferative index (Ki-67 index > 5%) (Figure 3) [59] and IGF2 over-expression to confirm the diagnosis of ACC [60].

A relatively new system published by a European group in 2015 is the Helsinki score which relay on mitotic rate, necrosis and Ki-67 index (3× mitotic count [>5/50 high power fields] + 5× presence of necrosis + Ki-67 proliferative index in the most proliferative index of the tumour) of ACC and focus on the predicting diagnosis as well as prognosis of ACC [61,62]. A Helsinki score >8.5 is associated with metastatic potential and warrant the diagnosis of ACC.

**Figure 4:**
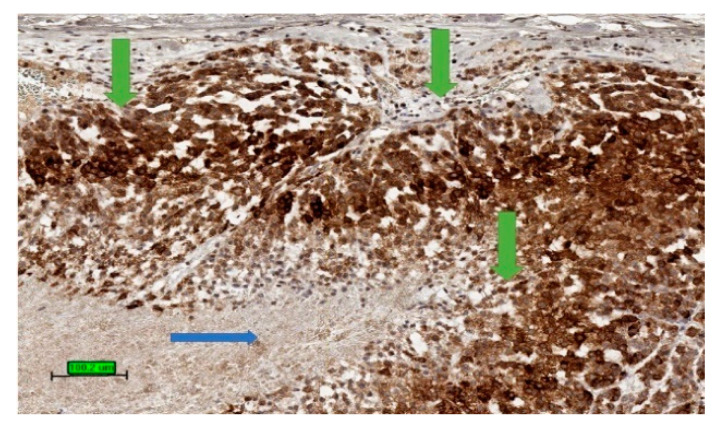
Tumour stain positive (brown colour) for inhibin (3,3′-diaminobenzidine/haematoxylin stains) in cytoplasm of ACC (green arrows). The necrotic area is not stained up (blue arrow). (scale bar—100 µm).

The most common malignant tumour in adrenal gland is metastatic carcinoma [63]. In a biopsy specimen, it is important to differentiate ACC from metastatic carcinoma as well as phaeochromocytoma by clinical history, biochemical studies, radiology, and a panel of immunohistochemical stains [64]. ACC expresses markers specific for steroid-producing cells which often include steroidogenic factor-1 (SF-1) and inhibin alpha (Figure 4). The tumour is also positive for markers expressed by other tumour types, such as melan A and calretinin. In addition, abnormal beta-catenin intracellular accumulation is often noted [15]. Common epithelial markers such as cytokeratin, EMA, CEA are generally negative. Although ACC could be positive for synaptophysin, it is negative for chromogranin.

Distant metastases at presentation occurred in approximately one third of the patients with ACC [15]. The common sites of distant metastases of ACC are liver, lung, and bone [65]. Unusual descripted metastatic locations of ACC have been reported in the stomach [66], pancreas [67], skin [68], spleen [69], tongue [70] and brain including meninges [71,72]. Recurrence and distant metastases of ACC often occur quickly (within 2 years). However, late locoregional recurrence could occur 18 years after surgery [73] and liver metastasis have been reported in a patient 23 years after surgical resection of ACC [74].

### 2.4. Prognosis

Prognosis of the conventional ACC depends on the cancer stage [12]. In addition, the prognostic value of Helsinki score has been demonstrated to be an outperforming prognostic index [56]. In addition, large tumour size and cortisol-secreting tumour were additional factors for ACC-specific death [49]. Moreover, there are many predictive features proposed for the prognosis of patients with ACC such as mitotic grading [52], Ki-67 index [75], mi-RNAs expression [76], expression of PDL-1 [77], SF-1 [78] and sterol-O-acyl transferase 1 (SOAT1) [79]. Preliminary data also shows that for localised ACC, molecular makers (expression, methylation, and chromosome alterations) could predict cancer recurrence [9]. Nevertheless, many of these markers need validation and some (such as molecular markers) are difficult to apply in clinical settings.

Overall, the prognosis of patients with ACC is poor. A recent Danish study on 160 patients with ACC showed that a median survival of patients with ACC was 35 months [14] whereas in the study based on the USA SEER database, the median survival of patients with ACC was 17 months [4]. The 5-year cancer specific survival rate of patients with ACC as noted from USA SEER database was 38% [1]. It is worth noting that surgery on the primary site even in metastatic ACC significantly improved overall and cancer-specific survival of patients with ACC [80]. The 5 year-survival of adult patients from multiple datasets with ACC after surgery range from 40% to 70% [49,81]. The estimated five-year overall survival rate for patients with ACC in recent cohorts is slightly less than 50% [11]

## 3. Paediatric Adrenocortical Carcinoma

### 3.1. Demographic Characteristics

ACC in paediatric patients deserves separate grouping as they are different from ACC in adults in terms of biological behaviour. The incidence of paediatric ACC is 0.2 cases per million children per year in SEER data from USA [82]. A recent national cohort study in France suggested the incidence of paediatric ACC to be approximately 0.1 cases per million children per year [83]. On the other hand, the incidence of paediatric ACC is reported to be high in southern regions of Brazil in which 4 case per million was reported in children of less than 10 years which may be related to high incidence of germline mutation of *TP53* (R337H) detected in the region [84,85].

ACC in paediatric group is more commonly seen in girls (male to female ratio of 1 to 2). Paediatric patients with ACC accounted for 6.5% of patients with ACC [4]. The disease is more common in the first decade with peak in 1 to 4 years [82] and with the mean age at presentation at 5 [86]. Infants of less than 6-months-old with ACC have been reported [87,88,89].

### 3.2. Clinical Features

Paediatric ACCs are often functioning tumours. In the largest single-institution study [90] involving 41 paediatric ACC, only 15% of the patients were non-functioning. In the functioning tumours, many secrete more than 1 hormones (54%) and the most common hormonal manifestation is related to sex hormones. The sex hormones produced by paediatric ACC are mostly androgen (virilising effects) [91] with a couple of paediatric ACCs oestrogen secreting [92].

A few paediatric ACCs occur in the setting of hereditary syndromes. They were found in Li-Fraumeni syndrome [87,93,94], multiple endocrine neoplasia type 1 [95], neurofibromatosis type 1 [96] and familial adenomatous polyposis [97]. In addition, paediatric ACCs were found in Beckwith–Wiedemann syndrome [98,99].

### 3.3. Pathology

ACC is often noted in the left adrenal gland in paediatric patients (left to right ratio = 1.4 to 1). The median size of paediatric ACC was 95 mm (range, 2 to 200 mm) [90,100].

Like adult ACC, paediatric ACC may sometimes need criteria to differentiate from cortical adenoma and borderline cortical tumour. Weiss criteria used for adult ACC is not applicable to paediatric tumours as it will label many tumours of benign behaviour as ACC. Thus, Wieneke and colleagues from the Armed Forces Institute of Pathology (AFIP) of the USA developed a set of criteria in 2003 and have tested on 83 paediatric adrenal cortical neoplasms in USA, for the purpose of predicting the behaviour of these tumours [101]. Wienke criteria comprises 9 parameters which incorporate macroscopic features (tumour weight > 400 g; tumour size > 105 mm) as well as microscopic features (extension into periadrenal soft tissues and/or adjacent organs, invasion into the vena cava, venous invasion (emboli, independent of main tumour), capsular invasion (beyond the capsule), tumour necrosis (confluent), atypical mitotic figures and high mitotic count (defined by more than 15 mitoses/20 high-power fields). Tumours with two or fewer criteria were cortical adenoma, those with 3 as “indeterminate” for malignancy and tumours with 4 or more criteria as ACC. The criteria were reported to be superior to Weiss by a few studies in the USA, Australia, and India [90,102,103,104]. Recently, in 2019, the criteria have been validated by Picard and colleagues in 95 paediatric adrenal cortical neoplasms from France [83]. In addition, the use of the Ki-67 index (>15%) was proposed to use to predict the outcome of paediatric patients with ACC.

### 3.4. Prognosis

Different from adult patients with ACC, paediatric patients with ACC often have better survival rates than adult patients with the disease [105]. Due to the small number of cases, studies in literature have shown different 5-year survival rates reported for paediatric patients with ACC, ranging from 34% to 100% [106]. The 5-year survival reported by Gupta and colleagues in a largest single-institution study on 41paediatric patients with ACC was 61% [90]. Recurrence was not detected in over 75% of the patients after treatment.

Approximately one third (31%) of paediatric patients with ACC presented with metastatic disease at the time of diagnosis [100]. Common sites of metastases are liver and lung [90]. Gulack and collages, in analysis 111 paediatric patients on USA national cancer database reported that age, size, extension of tumour, metastatic disease and margins status were associated with the survival of the patients with paediatric ACC [100]. Also, McAtter and colleagues based on 85 cases in SEER showed that older age and distant metastases were significant predictors of cancer-specific death [82]. Furthermore, Picard and colleagues showed that histological features in Wienke score (tumour necrosis, capsular invasion, venous invasion, high mitotic count) as well as high Ki-67 index are associated with worse outcomes [83]. In addition, a recent meta-analysis (published in 2021) on 42 studies with 1006 patients showed age, non-secreting tumours, complete surgical resection, small tumour (weight, volume and dimension) and low tumour stage are associated with better outcome [107]. Patients affected by Cushing syndrome showed a worse outcome.

## 4. Oncocytic Adrenocortical Carcinoma

### 4.1. Demographic Characteristics

Oncocytic adrenocortical carcinoma is defined as ACC with abundant oncocytic cytoplasm which is due to the accumulation of mitochondria. This variant was first mentioned in the third edition of WHO classification but without description of histological details [108]. Amongst the histological variants of ACC, oncocytic ACC is the most common variant. Different from other variants, oncocytic ACC often being studied together with other members of oncocytic adrenocortical neoplasms, namely adrenal oncocytoma and borderline oncocytic tumour.

In 2018, Kanitra undertook a systemic review on 140 adrenocortical oncocytic tumours with documented details reported in the literature and noted that 35% were benign, 41% were borderline and 24% were malignant (oncocytic ACC) [109]. Since the review by Kanitra, there are couples with oncocytic ACC and two large series of oncocytic adrenocortical tumours presented on the importance of Ki-67 expression and genomic profiles [110,111]. These 2 series do not provide data on the clinical pathological data of the individuals.

The unique features of oncocytic ACC have never been studied in separation from the oncocytic adenoma or borderline oncocytic neoplasm in the literature. In this review, all the features of documented oncocytic ACC were reviewed. The first case of oncocytic ACC was confirmed in 1991 by electron microscopy showing the closely packed mitochondria in an ACC from a 56-year-old man [112]. Up to the year 2020, there were 56 cases of oncocytic ACC in the adrenal gland with documented features in the English literature [112,113,114,115,116,117,118,119,120,121,122,123,124,125,126,127,128,129,130,131,132,133,134,135,136,137]. Apart from these cases, a giant ectopic oncocytic ACC (280 mm in greatest dimension; 2250 g in weight) have been reported in a 26-year-old Hispanic woman in tissue around the left adrenal gland and kidney {Wadhwani 33,281,927} [138].

In the WHO classification, the Lin–Weiss–Bisceglia (LWB) criteria proposed in 2004 is adopted to differentiate the different members of oncocytic neoplasms [117]. The Weiss score is not used as three of the criteria in the Weiss score—high nuclear grade, <25% clear cells, and diffuse architecture are characteristics of adrenal oncocytic tumour. In the LWB system, the presence of any one of the three major criteria—high mitotic rate (>5 mitoses per 50 high-power field), atypical mitotic figures and venous invasion—indicates the oncocytic adrenal tumour is the oncocytic variant of ACC. These criteria are amongst the nine features noted in the Weiss score [13]. Any of the minor criteria in the system in adrenal oncocytic cortical neoplasm (necrosis, sinusoidal invasion, capsular invasion, and large size/weight (size >100 mm and/or weight >200 g) indicates that it is of uncertain malignant potential.

According to the review by Kanitra, male patients with oncocytic adrenocortical tumour were more likely to be malignant [109]. It follows that oncocytic ACC do not show female gender predilection as in conventional ACC. From the pooled data in literature, the male to female ratio for oncocytic ACC is 1 to 1.1. The mean age at presentation of the patients with oncocytic ACC was 48 (range, 1 to 83), which is like that of conventional ACC [15]. On the other hand, the carcinoma is most often seen in younger patients in the fourth decade; with 30% of the cases reported in this age range. There are only 2 patients with oncocytic ACC in the paediatric group. They are both sex hormone producing oncocytic ACC; one is an 18-month body with androgen production tumour and co-existing rhabdomyosarcoma [137] and one is a 19-year-old female with testosterone secreting [124]. The oldest case of oncocytic ACC was noted in an 83-year-old female with non-functioning oncocytic ACC [130].

### 4.2. Clinical Features

In the literature, half of the documented cases of oncocytic ACC were functioning. The non-functioning cases most often presented with abdominal mass or pain as in conventional ACC. The most common presenting syndrome was related to the secretion of sex hormones, accounting for approximately half of the functioning cases. The other common functioning status was due to cortisol. Oncocytic ACC with Conn syndrome (aldosterone secreting) without other hormonal changes was reported in detail only in a 25-year-old man from Canada [121]. A patient with oncocytic ACC with multi-hormonal syndromes was associated with Lynch syndrome [133].

### 4.3. Pathology

From the pooled data of the 56 cases in the literature, oncocytic ACC occurs predominately on the left side with left to right ratio of 1.6 to 1. The mean and median maximum dimension of the reported case were both 130 mm. The size is slightly larger than the size range reported for conventional ACC (100 to 120 mm) as mentioned. The mean and median weight of the adrenal gland with the tumour was 829 g and 552 g respectively (range, 50 g to 5720 g). The largest oncocytic ACC was reported by Wong and colleagues in Australia who reported in their series an oncocytic ACC of maximum tumour dimension of 285 mm and with weight 5720 g in a 41-year-old woman [125].

**Figure 5:**
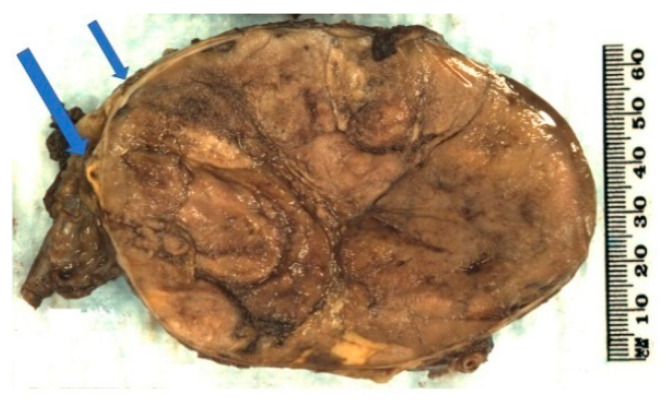
Oncocytic adrenocortical carcinoma showing tan lobular tumour on macroscopic examination. The tumour is rimmed by normal adrenal cortex (yellow colour, arrows).

**Figure 6:**
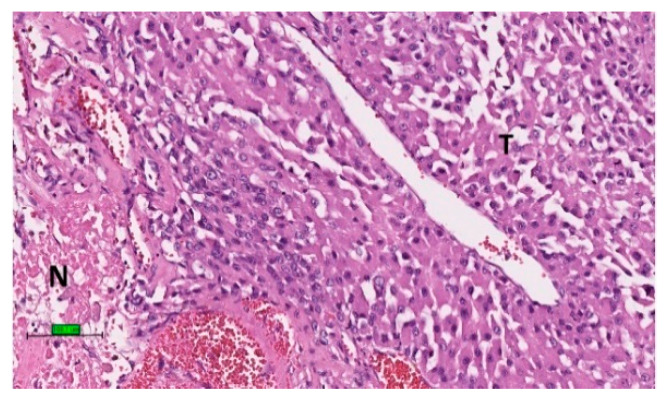
Oncocytic adrenocortical carcinoma: microscopic examination showing tumour cells with oncocytic (pink) cytoplasm (T) with necrosis (N) (haematoxylin and eosin stain) (scale bar—100 µm).

Macroscopically, in contrast to conventional ACC with yellow cut sections, the tumour is tan or brown on cut sections (Figure 5). Necrosis is common [134]. Microscopically, the tumour cells have abundant granular eosinophilic cytoplasm (oncocytes) and high-grade nuclear features (Figure 6). Intranuclear inclusions and frequent atypical mitotic figures were noted. The diagnosis of oncocytic ACC is often obvious after exclusion of adrenal oncocytoma and borderline neoplasm by LWB system. In addition, oncocytic tumours that are positive for synaptophysin and negative for vimentin are more often benign [109]. Myelolipomatous component have been demonstrated in a oncocytic ACC in left adrenal gland of a 69-year-old woman [125].

The immunohistochemical profiles of oncocytic ACC is like that of conventional ACC. Nevertheless, only half of the oncocytic ACC express alpha-inhibin and slightly more than one third of oncocytic ACC express melanin A [109]. Thus, a panel of markers should be used in case of doubt. Wong and colleagues recommended a strong, diffuse, and finely granular staining pattern of anti-mitochondrial antibody, mES-13, to document the oncocytic differentiation of the tumour [125].

The differential diagnoses could include malignant oncocytic neoplasms in the adjacent region such as from the kidney or liver. Rarely, oncocytic phaeochromocytoma could occur in the adrenal gland [139]. Chromogranin is important to differentiate them as unlike adrenal cortical oncocytoma, chromogranin have not been reported in oncocytic ACC but is always positive in oncocytic phaeochromocytoma [109,139].

### 4.4. Prognosis

Distant metastases were relatively uncommon for oncocytic ACC and documented in 7 cases: accounting for 13% of cases. The tumour could metastasise to liver, lung, bone, and ovary. Kanitra and colleagues reported that the 5-year overall survival rates for patients with oncocytic ACC was found to be 47% [109]. In addition, the pooled data from the literature revealed that median survival of 60 months for patients with oncocytic ACC. The prognosis appears to be better than conventional ACC and Helsinki score appear to predict the prognosis of the patients with oncocytic ACC [110].

## 5. Myxoid Adrenocortical Carcinoma

### 5.1. Demographic Characteristics

The myxoid variant of ACC is defined as ACC with abundant extracellular connective tissue mucin. In the 3rd edition of WHO classification, it was mentioned that ACC could have “myxoid changes” [108]. Only in the current 2017 WHO classification (fourth edition), it was formally labelled as a variant of ACC [13]. Myxoid ACC is an uncommon variant of ACC and first reported by Tang and colleagues in 1979 in a 41-year-old woman [140]. Up to the year 2020, 47 cases of myxoid ACC have been reported in the literature [140,141,142,143,144,145,146,147,148,149,150,151,152,153,154].

Unlike conventional ACC, myxoid ACC do not show gender predilection (24 males and 23 females). The mean age at presentation of patients with the tumour was 48 (range, 19 to 82) which is like that of conventional ACC [15]. The carcinoma is most often seen in the fifth decade. All the patients of myxoid ACC noted in the literature are adult patients. The youngest patient was noted in a 19-year-old girl [149].

### 5.2. Clinical Features

In the literature, 57% (*n* = 27) of the documented cases of myxoid ACC were functioning. The most common presenting syndromes were Cushing syndrome (*n* = 23), accounting for 49% of the documented cases. There were also 4 patients with Conn syndrome as well as 4 of the 23 patients with Cushing also had increased aldosterone secretions. No cases presented with sex hormone secretion. Recently, Harada and colleagues reported a functioning myxoid ACC in a 68-year-old Japanese woman with increased aldosterone and cortisol in the setting of MEN 1 [153]. Tang and colleague, who reported the first case of myxoid ACC, noted that the patient had parathyroid hyperplasia and pituitary tumour which may be a case with MEN 1 [140].

### 5.3. Pathology

The laterality of the myxoid ACC was not documented in many cases. Nevertheless, myxoid ACC appears occur in the left adrenal more commonly (Left to right ratio = 1.5 to 1). The mean and median maximum dimension of the reported case was 125 cm and 100 mm, respectively. The mean weight of the adrenal gland with the myxoid ACC was 707 g (range, 38.5 g to 3200 g).

Macroscopically, the tumour had a yellow-brown appearance with variegated appearance like that of conventional ACC. In addition, gelatinous myxoid areas were noted [148,151]. Microscopically, the tumour is characterised by the cords or trabeculae of tumour cells floating in stroma with diffuse pools or lack extracellular mucin which is positive for alcian blue. The staining pattern favour acid mucopolysaccharides of connective tissue type mucin [146]. A minority of cases also positive for mucicarmine or periodic acid Schiff stains. This contrasts with conventional ACC which may have focal myxoid changes in the stroma [147].

In the literature, 81% (38/47) of myxoid ACC documented the proportion of myxoid component in the ACC. The median proportion of the myxoid component was 30%. Some features of myxoid ACC, in contrast to conventional ACC, are trabecular/micro acinar growth pattern, small, uniform cell size, mild nuclear atypia and scant, eosinophilic cell cytoplasm [147]. Thus, the lack of diffuse growth pattern and nuclear atypia (two Weiss criteria) make the use of Weiss parameters problematic in the application to assess the malignant potential. In addition, the myxoid stroma make the identification of invasive areas (another Weiss parameter) difficult. Myxoid changes could occur in cortical adenoma though uncommon [148]. By applying Weiss parameters, 3 cases of borderline myxoid adrenal tumours have been reported [147,155]. One case developed local and peritoneal metastases and should be reclassified as myxoid ACC [147].

Lipometaplasia were reported in 21% (*n* = 5) of myxoid ACC [143,149,150,152]. The morphology is a reactive degenerative or metaplastic process in tumour cells which appears to be detected in myxoid ACC.

The immunohistochemical expression pattern of the ACC component is like that of conventional ACC in general. Neurofilament and CD56 have also been noted in the myxoid areas [147].

Due to the myxoid appearance, the differential diagnoses of the tumour in biopsy includes many tumours with myxoid stroma such as chordoma, myxoma, extra skeletal myxoid chondrosarcoma, lipoma, liposarcoma, benign or malignant nerve sheath tumours, myxoid leiomyoma, myxoid leiomyosarcoma, gastrointestinal stromal tumour and myxoid myxofibrosarcoma [152]. Besides, the differential diagnoses must include metastatic carcinoma, as in the settings of conventional ACC. Throughout examination of the resected tumour with immunohistochemical and clinicopathological correlations would be able to come to the correct diagnosis.

### 5.4. Prognosis

Distant metastases occur often in patients with myxoid ACC. In the literature, 68% (32/47) of the patients had documented the sites of metastases in myxoid ACC. The two commonest sites of metastatic myxoid ACC were in liver (*n* = 17) and lung/pleura (*n* = 16). Metastatic carcinoma was also reported in the bones (*n* = 4). An uncommon metastatic myxoid ACC to the brain was noted in a 63-year-old woman [142].

Myxoid ACC appears to be slightly more aggressive than conventional ACC [152]. Tumour recurrence was documented in 20 patients with myxoid ACC. The median time for recurrence after surgery was 4.8 months. On pooling the data from the reported cases of myxoid ACC in the literature, the median survival of the patients was 29 months and the longest survival reported for patients with myxoid ACC was 69 months [149]. Helsinki score (based on mitotic count, necrosis, and Ki-67 index) was proposed to be of value in predicting the prognosis of patients with myxoid ACC, but the cases were too few to perform specific survival analysis [61].

## 6. Sarcomatoid Adrenocortical Carcinoma

### 6.1. Demographic Characteristics

Sarcomatoid adrenocortical carcinoma is characterised by the presence of mesenchymal differentiation in additional to the carcinomatous component. The carcinoma is the least common variant of adrenocortical carcinoma. All the reported cases were reported as case reports. In the 2nd edition of the WHO classification and the 3rd edition of the WHO classification of endocrine tumours, the carcinoma is labelled as “carcinosarcoma” {WHOs} [108,156]. Sarcomatoid ACC is now recognised as a distinct histological variant of ACC in the current WHO classification [13]. The carcinoma appeared first in Japanese literature in 1987 [157] and in English literature in 1989 [158]. Up to the year 2020, 28 patients with sarcomatoid adrenocortical carcinoma were documented in the literature [15,152,159,160,161,162,163,164,165,166,167,168,169,170,171,172,173,174,175,176,177,178,179].

From pooled data in the literature, sarcomatoid ACC is slightly more common in females with female to male of 1.3 to 1. The mean age at presentation was 56 (range, 23 to 79) which is within the upper range of those reported in a large series of conventional ACC [15]. The carcinoma was most often seen in the sixth and seventh decades. No paediatric patient with sarcomatoid ACC was reported in the literature.

### 6.2. Clinical Features

Most of the patients presented with non-functioning sarcomatoid adrenocortical carcinoma. This contrasts with conventional ACC, in which approximately half of patients had functional tumour [57]. In the literature, symptoms of sex hormone production (virilization) was noted in a 29-year-old American women [160] and aldosteronism was present in a 79-year-old American woman (the oldest patient with the disease reported in the literature) [161]. In addition, a 69-year-old Japanese woman with bilateral sarcomatoid ACCs presented with symptoms and signs of hypoadrenalism likely because of destruction of the adrenal glands [175]. Other than these, patients with sarcomatoid ACC often presented with localized pain (abdominal pain, loin pain, flank pain, back pain, etc.) related to the mass effect of the big adrenal tumour (18/28; 64%). One fourth (7/28; 25%) of patients with sarcomatoid adrenocortical carcinoma had non-specific symptoms or incidental findings.

### 6.3. Pathology

Two of the patients with sarcomatoid ACC had bilateral tumours [174,175]. The carcinomas were slightly more commonly seen on the right side (right to left = 1.4 to 1). They are often big with the mean and median maximum dimension of 129 mm and 127 mm respectively (range, 55 to 240 mm). This is slightly larger than the size range reported for conventional ACC (100 to 120 mm) [14,49]. The mean weight of the adrenal gland with sarcomatoid ACC was 620 g (range, 20 to 6500 g).

Macroscopically, like other ACC, many of the sarcomatoid ACCs were yellow and necrotic [152,171,172]. The necrotic changes could be extensive and giving cystic appearance. In the literature, 6 of the 28 sarcomatoid ACCs had cystic appearance [159,160,163,164,176]. In addition, the adrenal gland in sarcomatoid ACC was often extensively replaced by the tumour [164,175]. The sarcomatoid area may stand out as white fleshy foci [173].

Microscopically, sarcomatoid ACC comprises a mixture of conventional ACC component mixed with sarcomatoid component. The sarcomatoid component of ACC is mainly composed of spindle tumour cells. Prominent nuclear pleomorphism, atypical mitotic figures and tumour giant cells could be identified. There is no definition of proportion of spindle tumour cells required for the diagnosis of sarcomatoid ACC. However, the spindle cancer cells may comprise 70% of the tumour [160]. On histological examination, especially in small biopsy examination, it may be difficult to differentiate sarcomatoid ACC with predominant spindle cell differentiation from retroperitoneal sarcoma, leiomyosarcoma or gastrointestinal stroma tumour. It is important to sample the tumour to identify the carcinomatous component of the tumour. The carcinomatous component of sarcomatoid ACC staining profiles is the same as the conventional ACC. However, the sarcomatous component may have lost or partial lost in immunoreactivity to melan A, inhibin and SF-1. Cytokeratins may be present in the spindle cell components.

There are cases reported to have heterologous elements. In the literature, 4 cases had a rhabdomyosarcoma component [159,160,166,169] and 3 had osteosarcoma component [161,167,176]. One of the 3 cases with an osteosarcoma component had a chondrosarcoma component and was noted in a patient presenting with aldosterone excess [161]. There were two ACC with an oncocytic component, but the tumours should be classified as sarcomatoid ACC because the presence of sarcomatoid component carries a more biological aggressive potential. These include the case reported by Thway and colleagues of a 45-year-old Afro-Caribbean man with oncocytic features in ACC and with sarcomatous metastases [169] as well as the case reported by Kao and colleagues of a 48-year-old American woman having ACC with an oncocytic carcinomatous component and primitive neuroectodermal-like features (perivascular clustering of tumour cells and pseudorosettes) [170].

Due to its rarity, no detailed molecular works on sarcomatoid ACC was noted. Papathomas and colleagues have demonstrated in 6 cases of sarcomatoid ACC, that dysregulation of Wnt/beta-catenin signalling pathway and p53 mutation were common [176]. In addition, the stem cell makers and epithelial mesenchymal transition (EMT) markers were noted.

### 6.4. Prognosis

Distant metastases occur often in patients with sarcomatoid ACC. In the literature, 75% (21/28) of the patients had documented the sites of metastases in sarcomatoid ACC. These metastases either occur at presentation or within a few months after surgery. It is worth noting that for conventional ACC, only approximately one third of cases had distant metastases at presentation. The most common site of metastatic sarcomatoid ACC was in the liver (*n* = 16). The second most common site was in the thorax (lung/pleura/mediastinum) (*n* = 8). The other sites could include spleen, brain, and heart [152,157,169].

Sarcomatoid ACC appears to be more clinically aggressive than conventional ACC. Recurrence of the cancer was often noted shortly after surgical resection. Most (approximately 80%; 13/16) of the documented recurrence occurs within 4 months after surgery. The median survival of reported cases from the pooled data in the literature was 7 months and the longest survival reported for patients with sarcomatoid ACC was 30 months.

## 7. Comparison of Different Types of Adrenocortical Carcinoma

Table 1 shows the comparison of features of different variants of ACC summarised from the literature. This highlights the clinical and biological differences between the different histological variants of ACC justifying dividing them into distinctive groups in research.

In general, ACC are more common in females and on the left side. It is well demonstrated that adrenal tumours are higher in incidence in women [180] which is likely to result from the gender difference in hormonal interactions involving complex adrenal, endocrine and neurocrine functions as well as variations in hormonal receptor sensitivity. In addition, the left adrenal gland is bigger and heavier than right adrenal gland [181]. Thus, ACC has higher chance to occur in the left adrenal gland because of having a larger volume of cortical tissue. The only exception is sarcomatoid ACC which is more common on right adrenal. The difference may be related to data bias by the small number of cases reported or some other unknown factors, which needs to wait for more cases reported to be analysed.

Sarcomatoid ACC appear to differ in many aspects from other variants of ACC in terms of patients’ more advanced age at presentation, predominately non-functioning tumours, predilection of right side, larger tumour size, higher rate of distant metastases and worse outcomes of patients.

Patients with myxoid ACC and sarcomatoid ACC had much higher rates of distant metastases than those with oncocytic or conventional ACC. If follows that the patients with sarcomatoid ACC had the worse prognosis. For patients with myxoid ACC, the prognosis is slightly worse than those with conventional ACC.

**Figure 7:**
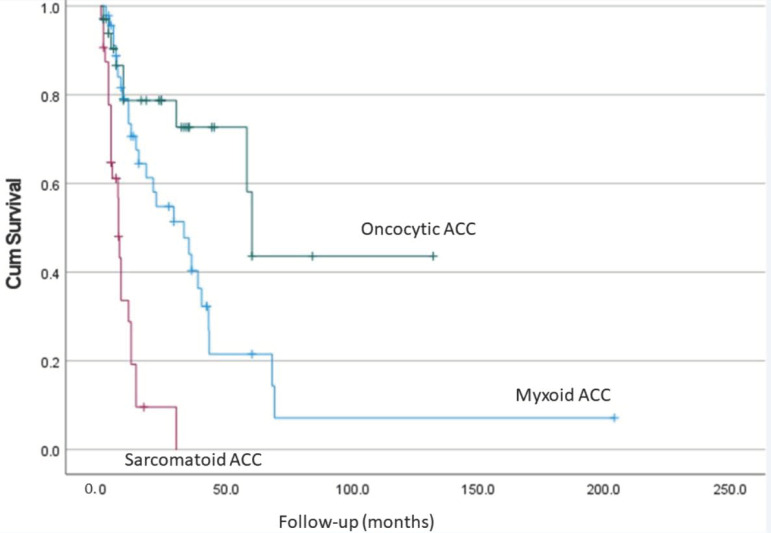
Survival analysis showing the difference in survival for patients with different types of adrenocortical carcinoma.

A plot of the pooled-up follow-up data available in the literature of all the cases of oncocytic, myxoid and sarcomatoid variants confirmed that patients with oncocytic ACC had the best prognosis (Figure 7). The prognosis of patients with oncocytic ACC is also better than that of prognosis of conventional ACC as noted from data in the literature (Table 1).

Renaudin and colleagues in France presented the largest series of oncocytic adrenocortical tumours in literature, also noted that patients with oncocytic ACC showed significant survival than those with conventional ACC [110]. It is worth noting that the findings concur that oncocytic ACC had lower mutation burden compared with the conventional or myxoid variants of ACC [111]. Renaudin and colleagues proposed that Helsinki score, which incorporates the Ki-67 proliferation index, was the most specific prognostic score for this group of adrenocortical tumours [110].

Genomic characterisation of ACC by next-generation sequencing published in 2014 to 2016 showed that ACC had multiple driver gene mutations [8,9,182]. Common mutated driver genes in ACC are *TP53*, *β-catenin* (*CTNNB1*), *IGF 2*, *zinc and ring finger 3 (ZNRF3)* and *telomerase reverse transcriptase (TERT)*. In addition, Pinto and colleagues had also identified genomic changes in paediatric ACC in 37 patients with ACC [7]. The driver genes mutations in paediatric ACC include *IGF-2*, *TP53* as well as mutations in *alpha-thalassemia/mental retardation, X-linked (ATRX), CTNNB1* and integration of human herpesvirus-6 in chromosome 11p. The authors noted that paediatric ACC could be divided into 3 groups based on *TP53* and *ATRX* mutations (group 1—*TP53* mutated and *ATRX* mutated; group 2—*TP53* mutated and *ATRX* wide type; group 3—*TP53* wide type and *ATRX* wide type) which may be associated with disease progression.

Molecular genomic characterisation of ACC on different types of ACC were studied by Vatrano and colleagues in a study in Italy [111]. The study noted difference patterns in gain and loss of copy number variations of common driver genes—*RB1, CDKN2A*, *ZNRF3*, *TERT*, *CDK4* between conventional, myxoid and oncocytic variants of ACC. In addition, p53/Rb1 pathway was the adverse molecular signatures associated with high tumour stage, high Ki-67 index, aggressive disease status and shorter disease-free survival.

## 8. Tumour Staging

The new 8th edition of the cancer staging manual of the AJCC adopted the information from European Network for the Study of Adrenal Tumours (ENSAT) [183]. Table 2 shows the details and the difference between the 7th edition and the current 8th edition of AJCC for ACC [7,184].

In the T-stage grouping for ACC, T1 is ACC ≤ 50 mm in greatest dimension with no extra-adrenal invasion; T2 is ACC more than 50 mm with no extra-adrenal invasion; T3 is ACC of any size with local invasion but not invading adjacent organs; T4 is ACC of any size that invades adjacent organs (kidney, diaphragm, pancreas, spleen, or liver) or large blood vessels (renal vain or vena cava). Invasion of large blood vessels (renal vein or vena cava) as thrombus, previously considered as M1 in AJCC 7th edition is now classified as T4. Overall, the only major change in the current prognostic group is to refine the division between Stage III and Stage IV. In the current prognostic staging, only ACC with distant metastases (M1) were classified as stage IV. In the 7th Edition of AJCC cancer staging manual, carcinoma of either T4 N0 M0 or T3/T4 N1 which was labelled as Stage IV in 7th Edition now re-classified into stage III. As noted for the discussion mentioned above, prognostic information like age, tumour grade based on mitotic counts, tumour size, functional status of the patients, tumour weight, vascular invasion, Ki-67 index and Weiss score were recommended information to be collected by the staging Manual. Furthermore, Poorman and colleagues in the USA (US adrenocortical carcinoma study group), based on 265 patients with ACC, have suggested the use of lymphovascular invasion as a criterion to better differentiate T2 and T3 but this option has not been validated [185].

Paediatric patients with ACC are different in biology from adult patients with ACC. The International Pediatric Adrenocortical Tumor Registry’s (IPACTR) proposed a modified tumour staging system and validated by the Children’s Oncology Group (COG) [186]. In the system, Stage I is ACC completely resected with negative margins, weight ≤100 g or ≤200 cm^3^, no metastasis; Stage II is ACC completely resected with negative margins, weight >100 g or >200 cm^3^, no metastasis; Stage III is residual or inoperable ACC and Stage IV is metastasis at presentation.

## 9. Pathological Reporting of Adrenocortical Carcinoma

With the refinement of WHO classification and pathological staging of ACC, standardization of pathological reporting of the disease is necessary for incorporation of the new information for the management and development of new therapeutics for this group of patients. Thus, shortly after the publication of new editions of the WHO classification and cancer staging protocol, the International Collaboration of Cancer Reporting (ICCR) developed a universal dataset for standardization of reporting for carcinomas of the adrenal cortex which was published on the website in 2019 [187]. Protocols for pathological reporting of adrenal gland tumours is not new and have been used in different countries such as Australia which developed a protocol of adrenal tumour in the year 2013 [188]. The ICCR is an international collaboration sponsored by major pathological bodies in the world. The expert group collected the protocols of different pathology groups in the world and modified into a universal dataset with updates of information from the new WHO classification and staging manual for adrenocortical carcinoma.

The ICCR dataset for adrenal cortex cancer comprises 23 core or required items to be available to clinicians after pathological assessment [187]. These core features include the classification according to WHO classification, integrity of the specimen, greatest dimension, weight, extent of invasion, architecture, percentage of lipid-rich cells, capsular invasion, lymphatic invasion, vascular invasion, atypical mitotic figures, coagulative necrosis, nuclear grade, mitotic count, Ki-67 proliferative index, margin status, lymph node status, and pathological stage. Tumours were dichotomized into low-grade (≤20 mitoses per 10 mm^2^) and high-grade (>20 mitoses per 10 mm^2^). It is worth noting that per mm^2^ have been used to replace high power field to standardise the size of high-power field under light microscopy. In the dataset, there are some other parameters (non-core) that have been proposed as well as the algorithms included for the diagnosis of ACC (Weiss, modified Weiss, Lin–Weiss–Bisceglia, reticulin, Helsinki and Armed Forces Institute of Pathology/Weineke).

Further modifications such as additional elements, values and commentary were adopted by local pathology societies such as in the Structured Reporting Protocol for Adrenal Cortical tumours by the Royal College of Pathologists of Australasia published in 2020 [189]. The later protocol provides additional information and explanations for adaption to be used of the local pathology communities. In addition, macroscopic cut-up manual for surgical specimen of adrenal gland is available to aid providing a proper reporting of ACC: (https://www.rcpa.edu.au/Manuals/Macroscopic-Cut-Up-Manual/Endocrine/Adrenal) (accessed on 1 February 2021).

## 10. Clinical Perspectives

Adrenal incidentalomas are common endocrine diagnoses which are adrenal masses that are discovered during radiological examination conducted for other reasons [190]. Often, they should be monitored in terms of radiological characteristics, clinical features, and functional levels. Functioning tumours traditionally will be managed by surgical approach. On the other hand, non-functional adrenal incidentalomas with no radiological features of malignancy and of small size will normally be monitored by radiological examination and functionally to see if there are any clinical progression [191]. An increase in size and development of functionality during follow-up in patients with adrenal incidentalomas will normally lead to biopsy and surgical resection. It is worth noting that computed tomography often underestimates the real size of adrenal lesions [192] so there is a tendency of lower threshold of the size of adrenal lesion for surgical management.

From information in Table 1, except for paediatric ACC, nearly half of the conventional, myxoid or oncocytic ACC are non-functioning and could present with incidentaloma. Furthermore, only 11% of the reported cases of sarcomatoid ACC are functioning. On the other hand, ACC accounts for less than 2% of adrenal incidentalomas [5,193]. The rate of malignancy depends on size of the tumour [190]. A study on 139 operations for adrenal tumour more than 50 mm in diameter showed that the most common tumour (around 25%) were ACC [194].

Studies used to establish historical guidelines of ACC have suffered from small sample sizes and use of low-resolution computed tomography and inclusion of purely surgical series [195]. The most recent recommendation for the optimal cut-offs for size and non-contrast computed tomography density to predict malignancy seems to be 46 mm and 20 Hounsfield unit (HU) (measurement of radio density) rather than 40 mm and 10 HU previously used [196].

Apart from ACC, silent pheochromocytoma was noted in 8.5% of adrenal incidentaloma in a national survey from Japan [5]. It is important to identify pheochromocytoma from ACC or other adrenal lesions by clinical, biochemical, and radiological means as biopsy is not indicated for pheochromocytoma because of safety considerations of life-threatening haemorrhage, hypertensive crisis and tumour implantation and death [197]. The main indications for percutaneous biopsy of adrenal lesions are ACC and metastatic malignancies [198]. Also, an endoscopic ultrasound-guided fine needle aspiration biopsy is a useful and safe technique to allow pathological and radiological diagnosis which is helpful in the detection rate of the non-functioning adrenal incidentaloma and such ACC, metastatic carcinoma, infection, etc., [199].

## 11. Conclusions

ACC is a disease of diverse clinical and pathological presentations. The prognosis of advanced ACC is still poor and novel treatments are needed. The results of most of the clinical trials of new drugs on ACC are disappointing [200]. In this review, the updates information of the classification, staging and pathological reporting of the tumour are foundations for understanding this heterogenous disease and testing of a new generation of therapies and management.

## Figures and Tables

**Table 1:** Characteristics of different types of Adrenocortical carcinoma.

Characteristics	Conventional		Oncocytic	Myxoid	Sarcomatoid
	Adult	Paediatric			
Number of cases	~8000	~200	56	47	28
Mean Age	47 to 55	5 (median = 4)	48	48	56
Most common age group (decade)	sixth/seventh	first (<5)	fourth	fifth	sixth/seventh
Male to Female	1 to 1.4	1 to 2	1 to 1.1	1 to 1	1 to 1.3
Functioning	half	85%	half	57%	11%
Most common hormone produced	Cortisol	Sex hormones	Sex hormones	Cortisol	-
Laterality	left adrenal	left adrenal	left adrenal	left adrenal	right adrenal
Left to right ratio	1.2 to 1	1.4 to 1	1.6 to 1	1.5 to 1	1 to 1.4
Bilateral	1%	-	none	none	7% (*n* = 2)
Size (median/maximum)	100–120 mm/280 mm	95 mm/200 mm	10 mm/285 mm	100 mm/300 mm	127 mm/240 mm
Weight (median [range])	528 g (38–4000)	276 g (20–1046)	552 g (50–5720)	450 g (38.5–3200)	620 g (20–6500)
Metastases	~ one third	31%	13%	68%	75%
	(26 to 35%)				
Median survival	17–35 months	-	60 months	29 months	7 months

**Table 2:** Comparison between 7th edition and 8th edition of American Joint Committee on Cancer (AJCC) in TNM prognostic stage grouping of ACC.

Prognostic Grouping	7th Edition	8th Edition
Stage I	T1 N0 M0	T1 N0 M0
Stage II	T2 N0 M0	T2 N0 M0
Stage III	T3 N0 M0	T3 N0 M0
	T1/2 N1 M0	T1/2 N1 M0
		T4 N0 M0
		T3/4 N1 M0
Stage IV	T4 N0 M0	
	T3/4 N1 M0	
	Any T N M1	Any T N M1

## Data Availability

All data from literature, analytic data available on equity on case to case base.
